# Comparison of analytic performances of Cellsearch and iFISH approach in detecting circulating tumor cells

**DOI:** 10.18632/oncotarget.6688

**Published:** 2015-12-19

**Authors:** Yuan Sheng, Ting Wang, Hengyu Li, Zhenzhen Zhang, Jianghao Chen, Chenyang He, Yongping Li, Yonggang Lv, Juliang Zhang, Cheng Xu, Zhen Wang, Chen Huang, Ling Wang

**Affiliations:** ^1^ Department of Vascular and Endocrine Surgery, Xijing Hospital, The Fourth Military Medical University, Xian, Shaanxi, China; ^2^ Department of Thyroid and Breast Surgery, Changhai Hospital, The Second Military Medical University, Shanghai, China; ^3^ Biotecan Medical Diagnostics Co., Ltd, Zhangjiang Center for Translational Medicine, Shanghai,China; ^4^ Department of Orthopedics, Xijing Hospital, Fourth Military Medical University, Xian, Shaanxi, China; ^5^ Department of Nephrology, Xijing Hospital, Fourth Military Medical University, Xian, Shaanxi, China

**Keywords:** breast cancer, circulating tumor cells (CTC), subtraction enrichment, cellsearch, aneuploidy

## Abstract

Circulating tumor cells (CTCs) have been widely used to predict the prognosis of breast cancer patients. The aim of the present study was to compare the performances of Cellsearch and immunostaining-fluorescence in situ hybridization (iFISH) in detecting CTCs in breast cancer patients. Forty-five newly diagnosed breast cancer patients and 14 healthy donors were recruited and their CTCs were detected by both Cellsearch and iFISH. Correlation between clinicopathological features and CTCs was investigated. We found that the positive rate of CTC detected by iFISH was significantly higher than by Cellsearch system (91% vs 38%). The CTC count, detected either by iFISH or Cellsearch, was not significantly associated with clinical pictures of patients with breast cancer. Therefore, we concluded that, compared to conventional Cellsearch CTC detection, in situ karyotypic identification performed by iFISH had higher detection rate. Therefore, iFISH may be more clinically useful than Cellsearch system.

## INTRODUCTION

Breast cancer is one of the most prevalent malignant cancer globally, with an annual incidence of 20 to 100 per 100,000 [1–4]. Although surgery and chemotherapy can improve the outcome of breast cancer, more than one-third of patients will suffer from relapse and die due to the metastasis [1, 2]. Predicting the prognosis and the risk of metastasis is a critical step in breast cancer management.

Circulating tumor cells (CTCs) are cancer cells that detach from primary or metastatic solid tumors into the vasculature, where they can be sampled from the circulating blood stream [5–7]. CTCs are commonly identified in the peripheral blood supply of diverse solid tumors, including breast cancer [8]. It has been reported that CTC detection was a promising tool for predicting the metastasis, as well as the prognosis of breast cancer [9–11].

Currently, Cellsearch system is the only approach approved by the United States Food and Drug Administration (US FDA) for detecting CTCs in patients with breast cancer [12]. The principal of this system is based on the epithelial cell adhesion molecule (EpCAM) on the tumor cell surface and cytokeratins (CKs) expressed in the same tumor cell. However, it has been reported that EpCAM is highly heterogeneously and dynamically expressed on many types of epithelial tumor cells, and epithelial-mesenchymal transition (EMT) may decrease the expressions of EpCAM and CKs and thus leads to the failure of CTC detection [13].

Recently, a novel CTC detection method named immunostaining-fluorescence in situ hybridization (iFISH) has been developed. Unlike Cellsearch system that depends on the cell surface markers, the iFISH system detects the abnormal chromosome content (e.g. chromosome) and proteins (e.g. PanCK, Vimentin, and HER2) located either on the cell surface or in the cytoplasm [14]. Previous studies have shown that iFISH had high CTC detection performance in patients with gastric cancer [15] or pancreatic cancer [16].

In this study, we studied the analytic performances of Cellsearch and iFISH in detecting CTCs in patients with breast cancer. To the best of our knowledge, this is the first report comparing the analytic characteristics of Cellsearch and iFISH CTC detection systems.

## RESULTS

### General data of subjects

The general data of the subjects were listed in Table 1.

**Table 1:** Clinical conditions of the subjects

	Breast cancer	Healthy	*P*
Sample size	45	14	--
Age (years)	54 ± 11	33 ± 18	<0.01
TNM stage (I/II/III/IV)	17/18/10/0	--	--
Estrogen receptor (Positive/Negative)	31/14	--	--
Progesterone receptor (Positive/Negative)	22/23	--	--
Cer-Bb-2 (Positive/Negative)	22/23	--	--
P53 (Positive/Negative)	29/16	--	--
Lymph node metastasis (Yes/No)	23/22	--	--

### Technical validation of iFISH detection approach

**Figure 1:**
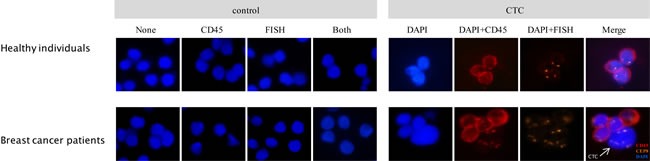
Detection of CTCs Cells from healthy donor blood and CTC revealed by subtraction enrichment combined with iFISH are shown (right). Cells from healthy donor blood were positive for CD45 and negative for CEP8 (upper-right). Arrow indicates aneuploidy CTC identified as DAPI+/CEP8+/CD45- (lower-right). Negative control and isotype control are shown (left). “None” indicates cells without any antibody staining or probe hybridizing, “CD45” indicates isotype control of CD45 antibody (mouse IgG2a), “FISH” indicates cells performed FISH without hybridization probe added, and “Both” indicates cells performed iFISH with isotype of CD45 antibody staining and no probe hybridizing.

Firstly, we evaluated the analytical performance of the iFISH method for CTC detection in breast cancer patients because it had not been reported in literature. Blood specimens from ten healthy individuals were set as negative controls. While no CTC was detected in these healthy subjects (Figure 1), CTCs were detected in breast cancer patients. Furthermore, we added 100 MCF-7, a well-known breast cancer cell line, into 7.5 ml of blood sample from a healthy individual. The recovery rate was 80% ± 7%, demonstrating that iFISH system was reliable for CTC detection in breast cancer patients.

### Positive rate of CTC in breast cancer detected by Cellsearch and iFISH

**Figure 2:**
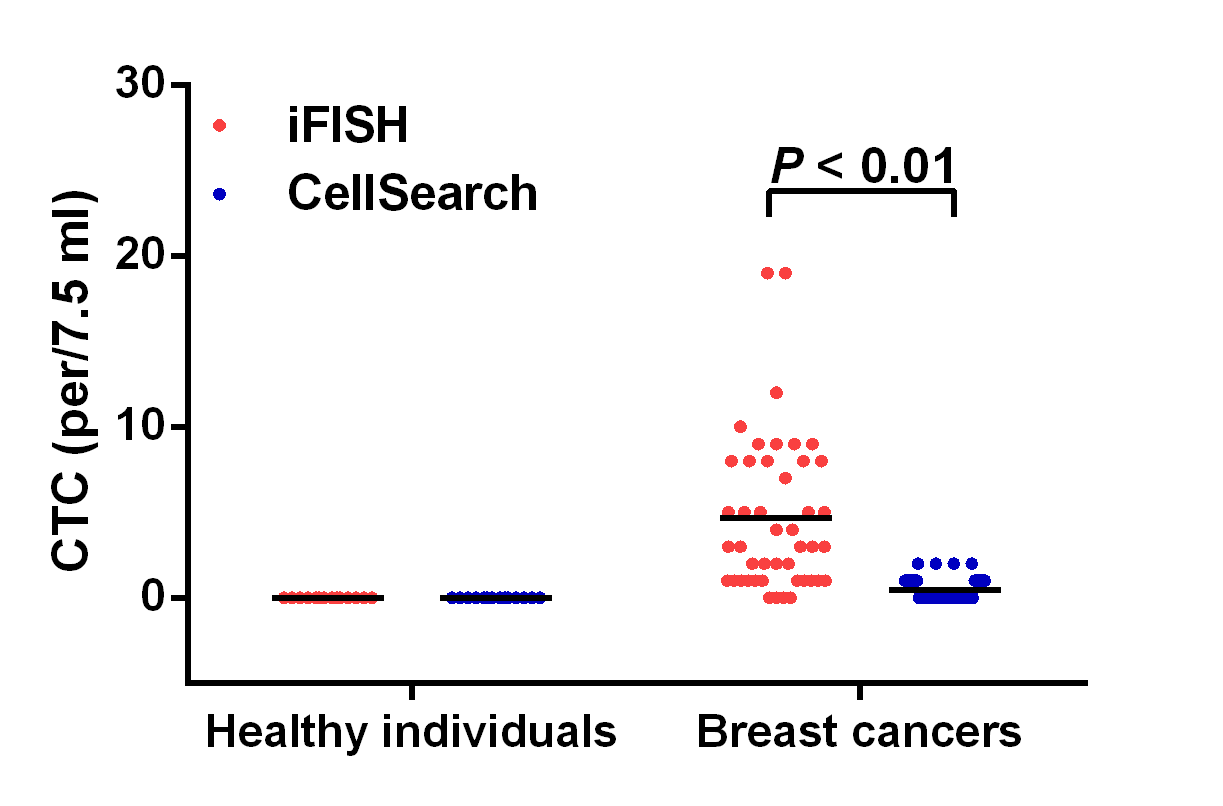
CTC distributions Horizontal lines represent the median values.

As shown in Figure 2, the number of CTCs detected was 0 - 2 /7.5 ml (median: 0/7.5 ml) for Cellsearch and 0 - 19 /7.5 ml (median: 3/7.5 ml) for iFISH. The CTC count detected by iFISH was significantly higher than that of Cellsearch (*P* < 0.01).

**Figure 3:**
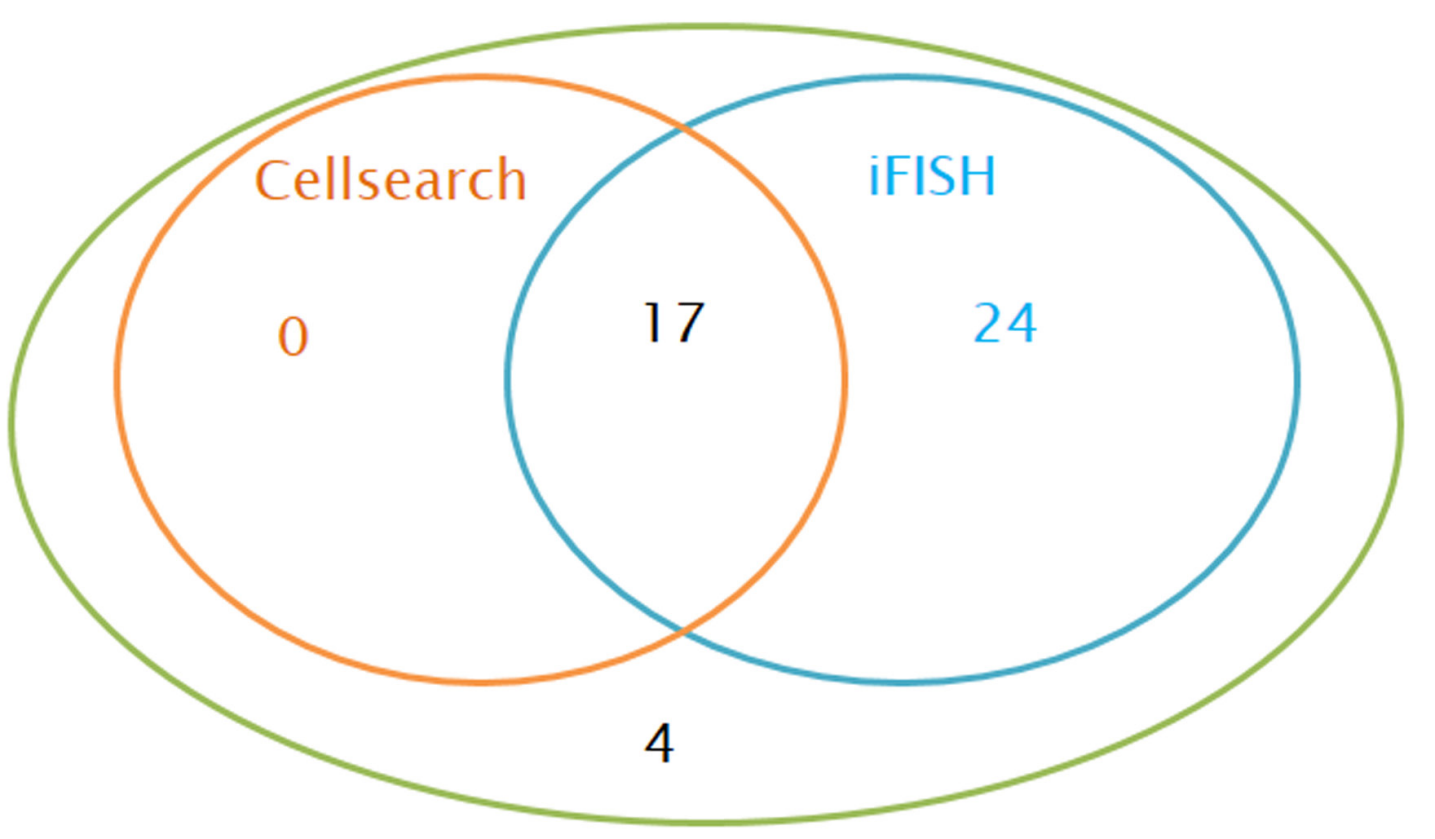
CTCs detected by Cellsearch and iFISH in patients with breast cancer ( ***n*** = 45).

As shown in Figure 3, CTCs were detected by iFISH in 41 of 45 patients (positive rate: 91%), showing a higher detection rate than that in Cellsearch (17 in 45 patients, positive rate: 38%). The kappa agreement coefficient between Cellsearch and iFISH was 0.11 (*P* = 0.10), indicating that the agreement between Cellsearch and iFISH was poor. In addition, we found that the relationship between the CTC count detected by Cellsearch and that detected by iFISH was not significant (*r* = 0.05, *P* = 0.73)

### CTCs and clinicopathological features

**Figure 4:**
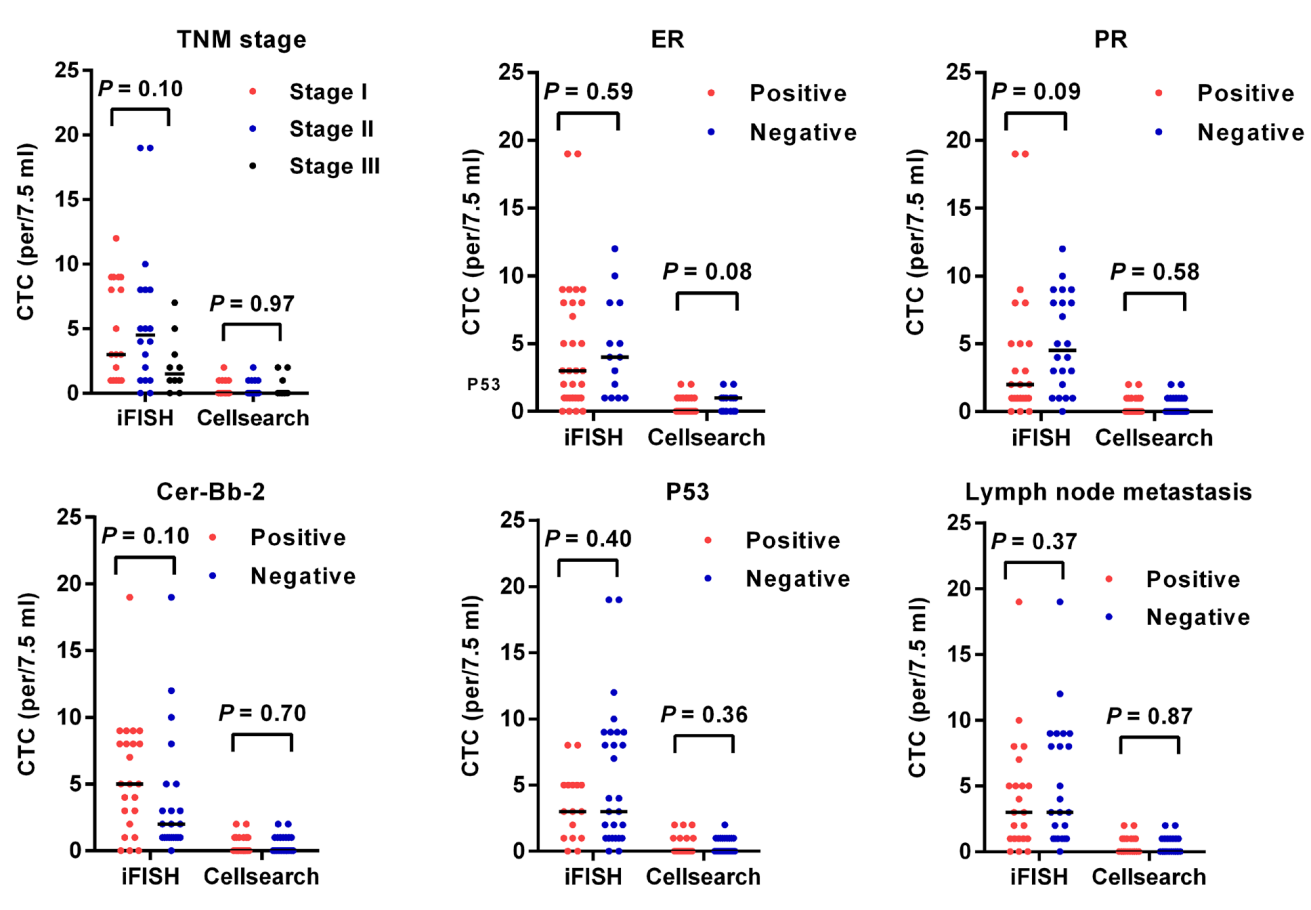
CTCs and clinicopathological features of patients with breast cancer Horizontal lines represent the median values.

Next, we analyzed the relationships between CTC count, either detected by iFISH or Cellsearch and the clinicopathological features of patients with breast cancer. Generally, no significant relationships were observed between CTC count and clinicopathological features (Figure 4).

## DISCUSSION

In the present study, we compared the analytic performances and clinical implications of Cellsearch and iFISH system in detecting CTCs. We found that the CTC count detected by iFISH was significantly higher than that detected by Cellsearch system, and the positive rate of CTC was markedly higher than that of Cellsearch. Thus, iFISH had higher detection rate than Cellsearch.

The higher detection rate may facilitate the application of iFISH in clinical practices. In this study, approximately two-thirds of patients with breast cancer were negative for CTC after detection with Cellsearch system. The prognosis of patients with negative CTCs may be heterogeneous, however, and their prognosis may not be well predicted by Cellsearch. By contrast, iFISH showed positive findings in 17 breast cancer patients with negative Cellsearch results . Thus, the prognoses of these 17 patients might be predicted by iFISH. Thus, the prognostic value of iFISH system may be more meaningful than that of Cellsearch. In addition, we noted that all the patients with positive Cellsearch findings were also positive after iFISH detection, indicating that the Cellsearch system may not be complimentary of iFISH.

The high positive rate of CTC detected by iFISH may be attributed to following reasons. The enrichment and detection of CTCs in Cellsearch system is cell surface marker (EpCAM)-dependent; however, the expression of EpCAM in breast cancer cells is heterogeneous and dynamic. During epithelial-mesenchymal transition (EMT), the expression of EpCAM on CTC may decrease [17]; therefore, these CTCs may be missed by Cellsearch system. By contrast, in the iFISH system, the cells were separated and enriched using CD45 magnetic beads; after the CD45-positive cells (i.e. the white blood cells) were removed, all the CD45-negative cells were kept for further identification. The enrichment process does not depend on the expression of CTC in certain markers and, therefore, is more sensitive.

We found that the CTC count, either detected by Cellsearch or iFISH, was not significantly associated with the patients’ clinical characteristics such as TNM stages, and lymph node metastasis. This is very interesting since it is well-known that these factors were strong prognostic factors for breast cancer. Thus, the prognostic value of CTCs, either detected by Cellsearch or iFISH, may not be overlapped with these factors. Indeed, many studies have found that CTC is a strong and independent prognostic factors for breast cancer independent of tumor stage, differentiation grade, and lymph node metastasis [18].

Our study had some limitations. First, the sample size in the present study was relatively small. Therefore, its conclusion needs to be validated by further studies with large sample size. Second, the subjects in this study were not followed and thus the prognostic value of CTC was not addressed. Further cohort studies were needed to explore the prognostic value of iFISH in detecting CTC in patients with breast cancer. However, to the best of our knowledge, this is the first study investigating the clinical implications of iFISH-detected CTC in patients with breast cancer, as well as the first study comparing the analytic characteristics of iFISH and Cellsearch CTC detection systems.

In summary, the iFISH CTC detection system has higher detection performance than that of the conventional Cellsearch system. Thus, iFISH represents a novel promising tool for predicting the prognosis of breast cancer patients.

## MATERIALS AND METHODS

### Patients and sample collection

A total of 45 patients with newly diagnosed invasive breast cancer and 14 healthy donors were enrolled at Changhai Hospital of Shanghai (China) from February 2014 to August 2014. Patients typically were presented with histologically confirmed invasive ductal carcinoma without advanced organ metastasis, although lymph node metastasis was present in some patients. Two 7.5-ml samples of peripheral blood were collected from each subjects prior to clinical treatment. Blood samples were separated by volume and stored in CellSaveTM Preservative tubes (Veridex, Raritan, 20 NJ, USA) for EpCAM+CTC detection by Cellsearch® system and an ACD tube (Becton Dickinson, Franklin Lakes, NJ, USA) for subtraction enrichment detection of aneuploidy CTC (iFISH).

The study was approved by the Ethics Committee of Changhai Hospital of Shanghai, and all participants provided written informed consent prior to participating.

### Subtraction enrichment of CTCs

Reagents for subtraction enrichment are provided by the Cytelligen CTC enrichment kit (Cytelligen, San Diego, CA, USA) according to the method suggest by Li et al. [15]. In brief, peripheral blood (7.5ml) was collected into ACD anticoagulant tubes. The supernatant was discarded after centrifuging the tubes within 48 hours after sample collection. Then, the sample was transferred to a centrifuge tube containing 3ml of the hCTC separation matrix. After centrifuging for 5min at 450 rpm, the cell suspension was collected from the buffy-coat layer. Immunomagnetic particles conjugated anti-CD45 antibody was added into the cell suspension, which was inoculated at room temperature for 10min and then placed on a magnetic stand (Promega, Madison, WI, USA) till the liquid became clear. The supernatant was pipetted off the magnetic field (non-magnetic bead-binding cell suspension) to remove leukocytes by centrifuging at 500 rpm for 2 min. The obtained cellular precipitation was immediately added with cell fixatives before smear making (cell fixatives and microscope slides were included in the reagent kit).

### Identification of aneuploidy CTCs

Reagents for CTC identification were provided by the Human Tumor Cell Identification kit (Cytelligen, San Diego, CA, USA). To identify aneuploidy CTCs, fluorescence in situ hybridization (FISH) and immunocytochemistry are used in combination. The cell smears were dried at 32 °C overnight ( > 4 hours). FR2 was preheated to 37 °C. After 20 μl of FR1 was mixed with 180 μl of FR2, it was immediately added into cell smears and let stand for 10min. After rinsing with FR3, the mixture was put into in a 100% alcohol after washing and let stand for 1min. After the slide was air-dried, 10 μl of probe solution containing fluorescence-labeled alpha-satellite probes for the centromeres of the chromosome (CEP8) (2μg/ml) was added and then covered with a coverslip and sealed with neutral resin. The hybridization procedure was as follows: degeneration at 75 °C for 5min, followed by hybridization at 37 °C overnight. Upon the completion of the hybridization, the slide was rinsed with FR3 and then added with monoclonal antibody anti-CD45 conjugated to Alexa Fluor 594 (Invitrogen, Carlsbad, CA, USA.) and anti-PanCK (CK4, 5, 6, 8, 10, 13 and 18.) (Invitrogen, Carlsbad, CA, USA) before inoculation at room temperature for 2 hours. Both of antibodies are 1:200 diluted. After rinsing with PBS, the slides were mounted with mounting medium containing DAPI and photographed with a fluorescence microscope (Nikon, Japan). CTCs were confirmed to be negative for CD45 and either positive for PanCK staining or aneuploidy chromosome 8.

### Detection of EpCAM-positive CTCs by Cellsearch system

The Cellsearch test was performed according to the manufacturer's instructions (Veridex LLC, San Diego, CA, USA) and the relevant literature [15]. Briefly, 7.5ml of peripheral blood was collected into the CellSave tubes containing EDTA and pre-prepared fixative. The samples were tested within 48 hours. After mixing with 6ml of the buffer, the blood sample was centrifuged at room temperature at 800 rpm for 10 min and then placed in the CellTracks Autoprep System. The EpCAM-positive cells in the samples were enriched using EpCAM-coated magnetic beads and then underwent immunofluorescence staining, which included the antibody cytokeratins (CK 8, 18, 19) conjugated to phycoerythrin, CD45 conjugated to allophycocyanin, and nuclear dye 4’, 6- diamidino-2-phenylindole (DAPI). Then, the cells were transferred to the CellTracks Analyzer II for scanning and analysis. In particular, the CK-positive and CD45-negative cells were the CTC (EpCAM^+^/CK^+^/CD45^-^).

### Statistical analysis

Statistical analyses were performed using SPSS 19.0 software (IBM, NY, USA). Positive rates were compared using Fisher's exact test. The agreement between iFISH and Cellsearch was determined by kappa test. Differences in CTC number between patients and healthy donors and among different CTC subtypes were compared by Mann-Whitney U test or Kruskal-Wallis H test. The correlation between CTCs detected by iFISH and Cellsearch was analyzed by Spearman approach. Graphical plots were generated using GraphPad Prism 6.0 (GraphPad Software, La Jolla, CA, USA). All the P values were two-sided, and a P values of less than 0.05 was considered statistically significant.
